# Subacute Thyroiditis Following COVID-19: A Case of Diagnostic Challenge in the Absence of Neck Pain

**DOI:** 10.7759/cureus.62203

**Published:** 2024-06-11

**Authors:** Kan Sakai, Tadahiro Yonaha, Takashi Shinzato, Takahiro Taira

**Affiliations:** 1 General Internal Medicine, Nakagami Hospital, Okinawa, JPN

**Keywords:** neck pain, thyroid peroxidase antibodies, thyroid complications, covid-19, subacute thyroiditis

## Abstract

This case report presents a 77-year-old woman who developed subacute thyroiditis following COVID-19. The patient exhibited atypical symptoms, including fever, fatigue, anorexia, significant weight loss, headaches, and palpitations, without the typical neck pain or tenderness associated with thyroiditis. One week later, a follow-up examination showed mild enlargement and tenderness of the thyroid. Laboratory tests indicated elevated thyroid hormone levels and suppressed thyroid-stimulating hormone. Ultrasonography revealed diffuse thyroid enlargement with poor blood flow, consistent with subacute thyroiditis. Despite the absence of typical neck pain, the diagnosis was supported by clinical, laboratory, and imaging findings. This case suggests the importance of considering subacute thyroiditis as a potential secondary condition following COVID-19, even in the absence of typical symptoms. Clinicians should consider that and perform thorough evaluations in patients with recent COVID-19 exposure and nonspecific symptoms.

## Introduction

In the era of the COVID-19 medical and health crisis caused by severe acute respiratory syndrome coronavirus 2 (SARS-CoV-2), many scientists have focused on the virus' detrimental effects on the respiratory system, including the lungs. However, the impact extends beyond these areas, affecting various bodily organs and systems such as the cardiovascular, gastrointestinal, genitourinary, and nervous systems [1].

Recently, several researchers have reviewed thyroid disorders linked to the endocrine system that manifest concurrently with or after COVID-19 infections [2,3]. Thyroiditis symptoms, such as neck swelling and discomfort, were noted during the peak phase of COVID-19 infection. Subacute thyroiditis is an inflammatory thyroid condition that typically follows infections due to viruses including SARS-CoV-2, and presents with excessive thyroid hormone levels [4,5].

Here, we report a patient with subacute thyroiditis following COVID-19 who presented without the typical thyroid pain or tenderness. Clinicians should consider subacute thyroiditis a potential secondary condition following COVID-19 infection and maintain a high index of suspicion, particularly in patients with recent COVID-19 exposure.

## Case presentation

A 77-year-old woman presented at our outpatient clinic of General Internal Medicine with a one-month history of persistent symptoms following COVID-19. Her chief complaints included fever (mainly nocturnal), fatigue, anorexia, and significant weight loss. She also reported headaches and palpitations and had been struggling with nausea and anxiety. Despite these symptoms, she denied experiencing night sweats, skin rash, itching, cough, sputum production, wheezing, chest pain, vomiting, dysphagia, diarrhea, hematochezia, melena, urinary frequency, polyuria, dysuria, polydipsia, and diaphoresis.

Her past medical history included heart disease (details unknown) and cervical spondylosis (no treatment), and she had no history of thyroid issues. She reported an allergy to mango but had no other known medication history. She had no significant family history and denied any history of alcohol or tobacco use.

On examination, her vital signs were blood pressure 108/52 mmHg, heart rate 105 beats per minute, respiratory rate 16 breaths per minute, body temperature 37.1℃, and oxygen saturation 98% while breathing ambient air. Her height was 156 cm, weight 51.2 kg (indicating a weight loss of 5.8 kg over the past month), body mass index was 21.0 kg/m², and Glasgow Coma Scale score of 15.

The patient appeared to be moving slowly and exhibited a postural tremor. The conjunctivae were not pale. Examination of the neck and supraclavicular regions revealed no lymphadenopathy or nodules. Her systemic examinations of the chest and abdomen were unremarkable. The skin was clear, and no rash or purpura was observed. Muscle strength of the extremities was 5/5, and the neurological examination revealed no focal deficits.

Her blood work-up, including complete blood count and biochemistry, was within normal limits except for the hemoglobin level (10.2 g per deciliter; reference range: 12.0 to 15.5 g/dL). Urine analysis showed ketones 3+, white blood cells were 10-19 per high power field, and a few bacteria, but no glucose, blood, or casts. An electrocardiogram showed a sinus rhythm with a regular rate of 90 bpm. A chest X-ray revealed no prominent infiltration or mass shadows.

Despite multiple symptoms, no significant findings were noted on physical and laboratory examinations. Given the presence of pyuria and bacteriuria and the elderly patient's lack of bladder irritation signs, it was considered asymptomatic bacteriuria. A tentative diagnosis of post-COVID-19 conditions was considered. An enhanced CT scan of the chest and abdomen was scheduled one week later to rule out malignancy.

**Figure 1:**
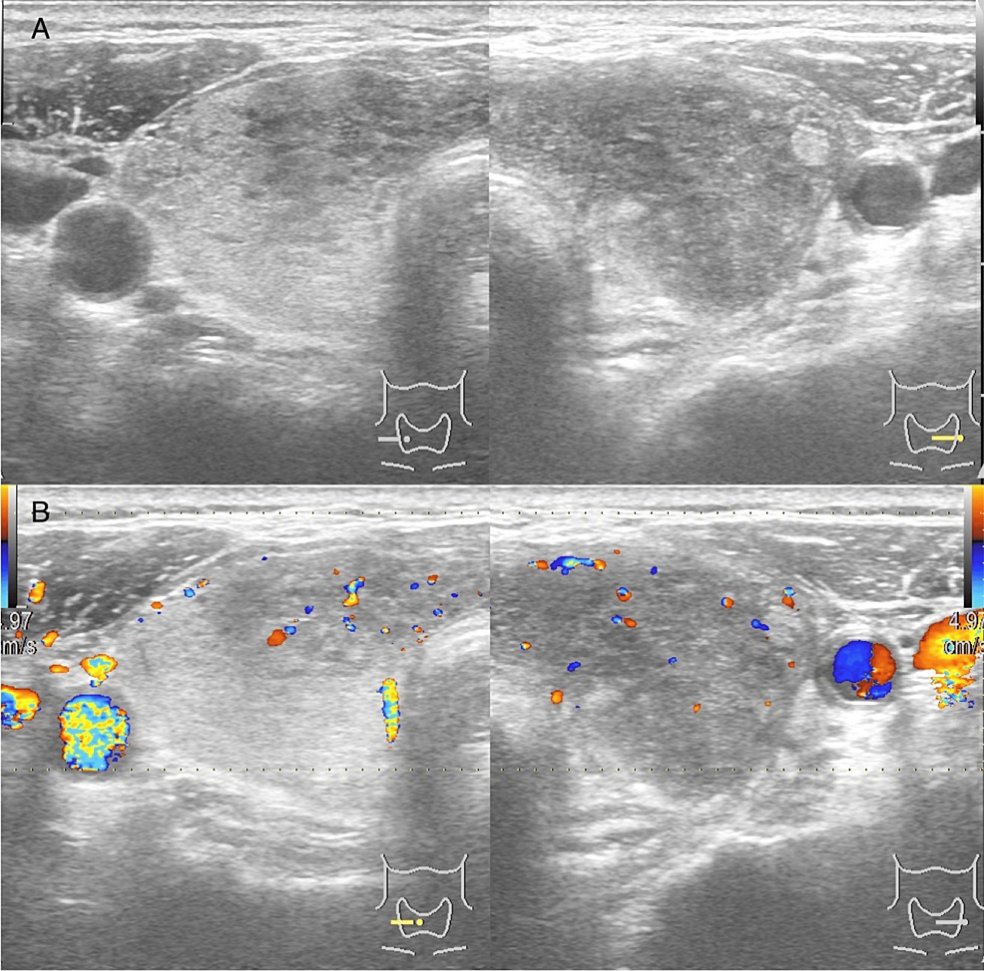
Thyroid ultrasonography and Doppler of the present case They revealed diffusely enlarged thyroid tissue with hypoechogenicity (A), and low blood flow (B).

On follow-up a week later, the patient continued to experience headaches and palpitations, with difficulty ingesting solid foods. Examination of the neck revealed a slight enlargement of the thyroid gland, which was mildly tender to the touch. The contrast-enhanced CT scan of the chest and abdomen showed no significant abnormalities except diffuse thyroid enlargement. Ultrasonography revealed diffuse thyroid enlargement with poor blood flow on color Doppler imaging and hypoechoic areas extending from the left lobe through the isthmus to the central part of the right lobe (Figures 1A-1B). Blood tests indicated elevated thyroid hormone levels with thyroid-stimulating hormone (TSH) <0.02 μU/mL (reference range: 0.27 to 4.20) and free T4 4.38 ng/dL (reference range: 0.9 to 1.8), suggesting a hyperthyroid state.

Despite low TSH and high free T4 values and the lack of typical thyroid tenderness, the ultrasound findings were consistent with subacute thyroiditis. Non-steroidal anti-inflammatory drugs were prescribed for headaches and β-blockers for tachycardia due to hyperthyroidism. One week later, the patient had significant improvement in headache and palpitations, and she was able to eat, leading to an increase in body weight. There was an increase in body weight. Further blood tests three weeks later showed that TSH and free T4 returned to normal ranges. However, thyroid peroxidase antibody (TPO) was positive, indicating the need for outpatient follow-up to monitor for potential hypothyroidism onset.

## Discussion

The etiology and pathogenesis of subacute thyroiditis have yet to be entirely understood. The Rochester Epidemiology Project reported the incidence of subacute thyroiditis to be 4.9 cases per 100,000 patients per year [6]. It is observed more frequently in younger females, with similar findings in Japanese studies [7].

Cases of subacute thyroiditis during or after COVID-19 have gradually increased in English literature [4,5]. SARS-CoV-2 is thought to cause subacute thyroiditis via a mechanism similar to other viral agents, including influenza, adenovirus, Coxsackievirus, Epstein-Barr virus, and cytomegalovirus [2-5]. A Korean study found the incidence of subacute thyroiditis related to COVID-19 to be higher, 17.3 per 100,000 patients [8]. A systematic review on subacute thyroiditis following COVID-19 showed a mean age of 42.7 years (range 18-85) and a predominance of women [4]. Our case involved an older woman, which was less common than the age group typically observed in previous studies.

The pathogenesis of subacute thyroiditis following COVID-19 remains incompletely understood. Two main hypotheses are proposed: direct viral invasion of thyroid follicular cells, triggering an immune response and subsequent thyroiditis; and a cytokine storm induced by the virus leading to nonspecific thyroid gland inflammation [3,9].

Silent thyroiditis, or painless thyroiditis, is an immune reaction involving the thyroid gland [10]. It can occur more often in women and can also be caused by medications such as interferon and amiodarone, tyrosine kinase inhibitors, and immune checkpoint inhibitors, which affect the immune system. COVID-19 may cause autoimmune thyroid disease or exacerbate underlying thyroid disease in remission [10,11]. In the present case, typical findings from laboratory and ultrasonographic examinations led to a diagnosis of subacute thyroiditis following COVID-19 despite no neck pain. However, we could not entirely exclude the possibility of silent thyroiditis due to an immune reaction between SARS-CoV-2 and the thyroid gland.

The diagnosis of subacute thyroiditis relies on clinical features, laboratory findings, and imaging. The disease exhibits characteristics typical of thyrotoxicosis and viral infections, including prodrome with myalgia, malaise, and fatigue [6,7]. COVID-19 can also cause prolonged sequelae and various symptoms, including general malaise, dysgeusia, dysosmia, low-grade fever, headache, and alopecia [12,13]. Neck pain is characteristic of subacute thyroiditis [6], while a systematic review on subacute thyroiditis following COVID-19 showed that 69% of patients had neck pain [4]. On the other hand, a few cases of painless thyroiditis after COVID-19 have also been reported [14-16]. In the present case, the absence of neck pain contributed to the diagnostic delay.

Some individuals develop hypothyroidism over time following subacute thyroiditis post-COVID-19. Previous studies revealed positive thyroid antibody levels of TPO, thyroid-stimulating immunoglobulin, and thyroid globulin receptor in some non-autoimmune thyroid diseases, suggesting that immunological hyperactivity in COVID-19 might lead to the formation of these antibodies [4,17,18]. A case of Hashimoto's thyroiditis developing after SARS-CoV-2 infection has been reported [19]. Our patient's laboratory data showed a positive TPO antibody, indicating the need for follow-up to monitor for the potential development of Hashimoto's thyroiditis.

This report aims to alert healthcare professionals about the potential for subacute thyroiditis following a COVID-19 infection. Subacute thyroiditis should be highly considered based on a characteristic medical history, symptoms, and results from blood tests and thyroid ultrasonography. In the absence of these distinctive presentations, diagnosis may be delayed. Physicians should inquire about symptoms and medical history in patients with indeterminate complaints following COVID-19 and examine in detail for signs such as rapid heart rate, enlarged thyroid gland with or without pain, tremors, and warm skin.

## Conclusions

Given the clinical resemblance between post-COVID-19 conditions and subacute thyroiditis, the thyroid axis may also be involved in COVID-19 or post-COVID conditions. Clinicians should consider that in patients with recent COVID-19 exposure.
